# Addressing disparities in delivery of cancer care for patients with melanoma brain metastases—Not just a simple case of rurality

**DOI:** 10.1093/noajnl/vdad113

**Published:** 2023-09-28

**Authors:** Joseph Riggs, Hyejeong Ahn, Hailee Longmoore, Pedro Jardim, Min J Kim, Ekkehard M Kasper, Jiali Han, Fred C Lam

**Affiliations:** Rural Health Scholarly Concentration, Indiana University School of Medicine, Terre Haute, Indiana, USA; Krieger School of Arts & Sciences, Johns Hopkins University, Baltimore, Maryland, USA; Faculty of Science, Clemson University, Clemson, South Carolina, USA; Faculty of Science, Providence College, Providence, Rhode Island, USA; Harvey Cushing Neuro-Oncology Laboratories, Department of Neurosurgery, Brigham and Women’s Hospital, Boston, Massachusetts, USA; Department of Neurosurgery, Saint Elizabeth’s Medical Center, Brighton, Massachusetts, USA; Faculty of Medicine, Boston University Chobanian and Avedisian School of Medicine, Boston, Massachusetts, USA; Department of Epidemiology, Richard M Fairbanks School of Public Health, Indiana University, Indianapolis, Indiana, USA; Harvey Cushing Neuro-Oncology Laboratories, Department of Neurosurgery, Brigham and Women’s Hospital, Boston, Massachusetts, USA; Department of Neurosurgery, Saint Elizabeth’s Medical Center, Brighton, Massachusetts, USA; Faculty of Medicine, Boston University Chobanian and Avedisian School of Medicine, Boston, Massachusetts, USA

Care for patients with brain metastases (BM) is complex given the specific treatment modalities required to address the spectrum of neurological and associated symptoms, notwithstanding the arduous management of their primary extracranial site of disease.^1^ Multidisciplinary teams involving neurosurgeons, neuro-oncologists, radiation oncologists, physical therapists, and other allied health professionals are essential to support these patients throughout the course of their illness.^2^ Whilst precision cancer therapies have dramatically improved the control of primary lung, breast, and melanoma cancers (the top three causes of BM),^3^ the inability of many of these therapies to gain access across the blood-brain barrier has led to an increased incidence of developing distant BM, which once diagnosed, have a 1- to 2-month median overall survival (OS).^4^ The incidence of developing BM in melanoma patients in particular have traditionally been reported to be as high as 50% with a dismal median OS of less than 5 months^5^; however, this has been greatly increased up to approximately 21 months with the combination of surgery, immunotherapy (IT), targeted therapies against BRAF V600E tumor mutations, and stereotactic radiosurgery (SRS).^6^ We recently reviewed the global consensus guidelines for the treatment of patients with melanoma BM (MBM) and concluded that while there is a global consensus recommending upfront combination IT or targeted therapies with or without SRS for the treatment of MBM, certain countries such as Japan have reported disparities in treatment patterns and patient outcomes due to a variety of reasons, ranging from the geographic distance to a major cancer center to access to insurance plans that covered these costly multimodal therapies.^7^ In this editorial, we aim to address the current disparities in delivery of care to MBM patients in the United States as a case study to begin to address the larger issue of increasing treatment disparities for brain tumor patients on a more global scale.

Recent 2017 statistics from the Surveillance, Epidemiology, and End Results (SEER) database reported an increased all-cancer incidence in rural (460 cases per 100 000 people) compared to non-rural (447 cases per 100 000 people) US counties.^8^ Approximately 97% of US landmass is considered rural, with 19.3% of Americans reported to be living in rural counties.^9^ According to the American Society for Clinical Oncology’s (ASCO) State of the Oncology workforce, 66% of rural counties lack an oncologist with 4 out of 10 current or previous cancer patients living in rural areas stating that they did not have access to an oncologist near their home. Rural areas have fewer specialists, more spread-out healthcare systems, and fewer hospitals or treatment centers.^10^ This is in contrast to 20 times more oncologists per square mile residing in urban areas.^11^ In terms of mortality from all cancers, metropolitan counties had a mortality rate of 166 deaths per 100 000 people while rural counties had a mortality rate of 182 deaths per 100 000 people. Rural patients also face a higher travel burden than urban-based patients who have easier access to academic centers by nature of urban proximity. A regional analysis of travel time to specialized health centers revealed that average travel times were increased across all levels of care for suburban populations (Table 1).^12^ Patients living in rural areas were 27% less likely to receive treatment at academic centers than their urban counterparts and had poorer overall survival compared to patients treated at academic facilities.^13^ However, a breakdown of survival of MBM patients treated at an academic center demonstrated an odds ratio of 1.02 which did not reach statistical significance (*P* = .67), suggesting that significantly prolonged OS of patients with MBM may hinge on other factors besides access to academic centers in metropolitan locations.^13^

**Table 1.** Analysis of Average Regional Travel Times for Cancer Patients Across All Levels of Care

	Median Travel Time		
Rurality	NCI Cancer Center	Academic-Based Care	Any Specialized Care
Urban	57 min (22–144)	22 min (11–46)	11 min (6–18)
Suburban	146 min (95–228)	83 min (56–125)	41 min (26–59)
Large town	168 min (107–254)	97 min (69–139)	51 min (38–70)
Small town or isolated area	180 min (118–260)	105 min (76–153)	59 min (43–80)

Average travel times in minutes (range of travel times listed in parentheses), from patient’s primary geographic location of residence across all levels of care. Rurality groupings were assigned by linking zip codes to a level in a 4-tier Rural–Urban Commuting Area (RUCA) classification system. The RUCA system is based on US Census data.

A study of melanoma patients living in Utah reported that patients living in metropolitan counties had a 92.7% 5-year median survival compared to a 91.5% in rural counties (survival difference rate of 1.2 favoring patients in metropolitan counties), suggesting that proximity to a metropolitan area affords melanoma patients with a survival advantage. One may argue that patients who do not live close to a major academic center should still be able to receive treatment closer to home from a local oncologist. However, in a study of cancer patients living in rural Appalachia where cancer incidence rate declines were similar to metropolitan and rural areas, the cancer mortality rate declines were less in rural areas. Rural residents had the highest cancer incidence rates as well as cancer mortality rates. For cancer sites combined rural residents had 5-year relative survival that was 5.2% lower than metropolitan residents. Rural residents experienced lower survival rates for brain cancer (6.6% lower). Rural residents were also more likely to have no radiation/surgery and have missing data for treatment. This study supports our postulate that even if there were to be access to local treatment centers, rural residents with brain cancer may not derive a survival benefit.^14^

Immune checkpoint inhibitors such as nivolumab and ipilimumab have significantly increased OS for patients with MBM^15^; however, data suggests that IT is 16% less likely to be given to cancer patients with lower socioeconomic status who are either uninsured or underinsured.^16^ Underinsurance may be associated with delays in surgery and treatment at low-volume centers. Insurance status is also associated with the stage of diagnosis, with uninsured patients presenting at more advanced disease stages compared with privately insured patients. In a study of 167 130 patients, 52% had commercial insurance, 43% had Medicare, 3% had Medicaid and 2% were uninsured. In patients below 65 yo, those with Medicaid and the uninsured had higher likelihood of presenting with metastatic melanoma and were less likely to receive IT compared with those with commercial insurance. Among those who received IT, patients with Medicaid (HR: 1.51, *P* = .001) and no insurance (HR:1.37, *P* = .046) had an inferior OS. In patients 65 years old and above, whereas Medicare was associated with an increased likelihood of presenting with metastatic disease, there was no significant difference in receipt of IT or OS as compared with commercial insurance. Insurance was associated with stage at diagnosis, receipt of IT, and OS for patients below 65 years old with melanoma.^17^ Under the Affordable Care Act, State Medicaid expansion status may improve the odds of lesser insured patients to receive IT.^18^ However, uninsured rural residents living in States that did not expand Medicaid under the Affordable Care Act are unable to access this benefit, creating a coverage gap for patients whose income is too high to qualify for Medicaid but too low to be able to afford marketplace insurance plans.^19^ These observations would suggest that insurance status affects disparities in treatment of patients with metastatic melanoma independent of rurality and socioeconomic status.

Ethnic differences may also contribute to treatment disparities. Patients of Hispanic or Asian decent are at higher risk of harboring BM than White patients at the time of diagnosis of their primary disease,^20^ and clinical trials evaluating the effectiveness of IT in Hispanics have demonstrated lower response rates and higher immune-related adverse events.^21^ A higher percentage of African Americans present with distant-stage disease with consistently lower OS^22^; however, data suggests that patients of Black race have better OS compared to White patients, suggesting that if access and quality of care is improved, Black patients might have a comparable or even better survival than White patients.^13^

**Figure 1.**
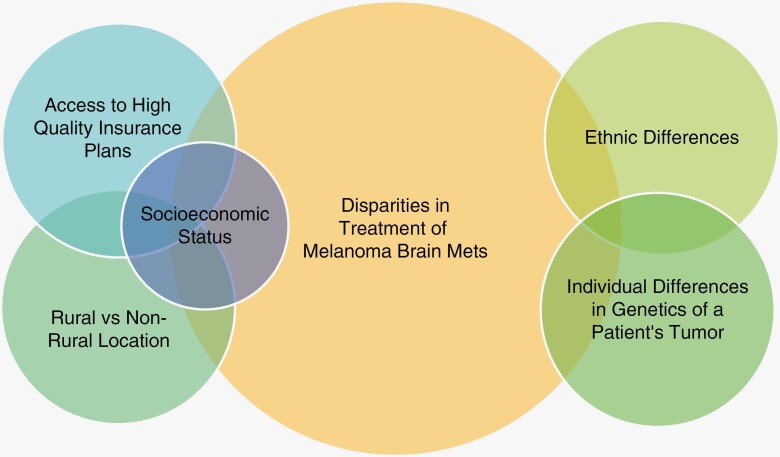
Venn diagram of causative factors contributing to disparities in treatment for patients with melanoma brain metastases. The left side of the Venn diagram demonstrates how rurality, socioeconomic factors, and insurance access affects disparities. The right side of the diagram alludes to racial, ethnic, and genetic differences that may contribute to treatment disparities.

Finally, a real-world treatment outcomes analysis of an NCDB data set comprising 3008 cases of MBM patients comparing IT versus multimodal combination therapies employing a Propensity Score Matching algorithm concluded that sex, year of diagnosis, income percentile, zip code of residence, use of chemotherapeutic agents, and IT alone versus IT + RT were *not* statistically significant in affecting patient survival. Rather, age, the presence of extracranial disease, the Charlson-Deyo comorbidity score, type of insurance, and the type of medical institution had statistical significance in patient survival.^18^ These data demonstrate that disparities in the treatment of MBM arise from 3 main factors: Accessibility to a medical system capable of managing a complex condition that is often compounded by various underlying conditions, an inequitable financial support structure provided by the insurance system; and preexisting medical conditions that negatively affect response to current treatment modalities. Given the complexities of issues governing the disparities in the delivery of cancer care to patients with MBM (Figure 1), it behooves us as a community to continue to strive for policy and advocacy change in the global healthcare landscape to achieve effective cancer care for all.
